# Pomegranate Extract Enhances Endothelium-Dependent Coronary Relaxation in Isolated Perfused Hearts from Spontaneously Hypertensive Ovariectomized Rats

**DOI:** 10.3389/fphar.2016.00522

**Published:** 2017-01-04

**Authors:** Nathalie T. B. Delgado, Wender do N. Rouver, Leandro C. Freitas-Lima, Tiago D.-C. de Paula, Andressa Duarte, Josiane F. Silva, Virgínia S. Lemos, Alexandre M. C. Santos, Helder Mauad, Roger L. Santos, Margareth R. Moysés

**Affiliations:** ^1^Department of Physiological Sciences, Centre of Health Sciences, Federal University of Espirito SantoVitoria, Brazil; ^2^Faculty of Pharmaceutical Sciences of Ribeirão Preto, University of São PauloRibeirão Preto, Brazil; ^3^School of Medicine of Ribeirão Preto, University of São PauloRibeirão Preto, Brazil; ^4^Department of Physiology and Biophysics, Federal University of Minas GeraisBelo Horizonte, Brazil

**Keywords:** hypertension, pomegranate extract, phytoestrogens, antioxidant effects, vasodilation, coronary arteries

## Abstract

Decline in estrogen levels promotes endothelial dysfunction and, consequently, the most prevalent cardiovascular diseases in menopausal women. The use of natural therapies such as pomegranate can change these results. Pomegranate [*Punica granatum L.* (Punicaceae)] is widely used as a phytotherapeutic agent worldwide, including in Brazil. We hypothesized that treatment with pomegranate hydroalcoholic extract (PHE) would improve coronary vascular reactivity and cardiovascular parameters. At the beginning of treatment, spontaneously hypertensive female rats were divided into Sham and ovariectomized (OVX) groups, which received pomegranate extract (PHE) (250 mg/kg) or filtered water (V) for 30 days by gavage. Systolic blood pressure was measured by tail plethysmography. After euthanasia, the heart was removed and coronary vascular reactivity was assessed by Langendorff retrograde perfusion technique. A dose-response curve for bradykinin was performed, followed by L-NAME inhibition. The protein expression of p-eNOS Ser^1177^, p-eNOS Thr^495^, total eNOS, p-AKT Ser^473^, total AKT, SOD-2, and catalase was quantified by Western blotting. The detection of coronary superoxide was performed using the protocol of dihydroethidium (DHE) staining Plasma nitrite measurement was analyzed by Griess method. Systolic blood pressure increased in both Sham-V and OVX-V groups, whereas it was reduced after treatment in Sham-PHE and OVX-PHE groups. The baseline coronary perfusion pressure was reduced in the Sham-PHE group. The relaxation was significantly higher in the treated group, and L-NAME attenuated the relaxation in all groups. The treatment has not changed p-eNOS (Ser^1177^), total eNOS, p-AKT (Ser^473^) and total AKT in any groups. However, in Sham and OVX group the treatment reduced the p-eNOS (Thr^495^) and SOD-2. The ovariectomy promoted an increasing in the superoxide anion levels and the treatment was able to prevent this elevation and reducing oxidative stress. Moreover, the treatment prevented the decreasing in plasmatic nitrite. We observed a reduction in total cholesterol and LDL in the Sham-PHE group. The treatment with PHE enhances the endothelium-dependent coronary relaxation and improves cardiovascular parameters, which suggests a therapeutic role of PHE.

## Introduction

Cardiovascular diseases (CVDs) are the main cause of death in many nations. Characteristics such as age, sex and genetic predisposition can be highlighted among the “non-modifiable” risk factors; whereas diet and lifestyle are considered “modifiable” (Leifert and Abeywardena, 2008; Mozaffarian et al., 2016).

Hypertension has been recognized as a major risk factor for CVDs (Pickering, 1972). However, studies have shown that premenopausal women have less risk of development hypertension and other CVDs when compared to postmenopausal women, suggesting vascular protective effects of female sex hormones (Farhat et al., 1996). The beneficial vascular effects of estrogen have been ascribed to a variety of factors, including endothelium-dependent and independent vascular relaxation (Gisclard et al., 1988). Furthermore, estrogen can reduce the oxidative stress, an effect that might be attributed to its capacity to decrease reactive oxygen species (ROS). However, the role of sex hormones (mainly estrogen) on the enzymes involved in the intracellular signaling, i.e., kinases, is very controversial. Indeed, while some authors demonstrated that estrogen could modulates kinases proteins expression such as Akt (Bhuiyan et al., 2011; Turdi et al., 2015), PKG (Word and Cornwell, 1998) and PKC (Hunter and Korzick, 2005; Hunter et al., 2007), others suggest that there is no modulation by estrogen on these kinases (Teede et al., 2001; Cao et al., 2015; Leung et al., 2015). Taken together, these studies showed that the relationship between the expression of kinases and estrogen levels is very complex which becomes a limiting factor for the use of estrogen therapy in postmenopausal women. In addition, the risk of uterine and breast cancer is another factor that restricts the use of estrogen therapy (Santos et al., 2014). Thus, new substances that could offer similar benefits as estrogen can be used as an alternative treatment in CVDs prevention (Borrás et al., 2010). Among them, pomegranate that has a high concentration of phytoestrogens has been used as hormonal therapy to reduce the postmenopausal symptoms (Glazier and Bowman, 2001; Auerbach et al., 2012). Even though some studies have shown beneficial effects of pomegranate, further research is needed.

The phytoestrogens are exogenous agents that have the ability to mimic endogenous hormones and these compounds are present in a wide variety of plants (Larrosa et al., 2006). The phytoestrogens have attracted attention due to its ability to act on the estrogen receptor (ER) subtype ERα, ERβ (Zhai et al., 2001; Papoutsi et al., 2005) and G protein-coupled oestrogen receptor (GPER) (Dong et al., 2013). Phytoestrogens may have beneficial effects on the cardiovascular system and also for osteoporosis and breast cancer (Lissin and Cooke, 2000).

*Punica granatum L.* (Punicaceae) is the fruit with the highest phytoestrogen concentrations, which contains significant amounts of phenolic antioxidants (Espín et al., 2007). This fruit is a native shrub of occidental Asia and Mediterranean Europe, popularly referred as pomegranate (Vidal et al., 2003). For centuries this plant has been used to ameliorate several diseases (Gracious Ross et al., 2001). Ancient Egyptian culture regarded the pomegranate fruit as a symbol of ambition and prosperity, making it common practice to decorate sarcophagi with this plant. According to ancients Ebers’ papyrus (1500 BC), the plant was used by Egyptians as a treatment for tapeworm and other parasitic infestations. The pomegranate, that was lauded in ancient times in the Old Testament of the Bible, the Jewish Torah, and the Babylonian Talmud is also known as a sacred fruit that conferring powers of fertility (Jurenka, 2008). In recent decades, pomegranate has been studied for many potential uses including: immunomodulation, atherosclerosis/arteriosclerosis, bacterial infection, fungal infection, parasitic infection, periodontal disease, and food poisoning (Gracious Ross et al., 2001; Prashanth et al., 2001; Schubert et al., 2002; Longtin, 2003). Currently, Sreeja et al. (2012) indicate that the pomegranate has a potential application as a possible alternative to hormone replacement therapy.

There has been great interest in pomegranate as a medicinal and nutritional product mainly due to its high polyphenol content, which contributes to a strong antioxidant effect, making it a useful tool against damage caused by hypertension (Mohan et al., 2010). Nevertheless, the effects of pomegranate on vascular reactivity of coronary bed have not been fully elucidated. Thus, we hypothesized that treatment with PHE enhances the endothelium-dependent coronary relaxation and improves cardiovascular parameters in ovariectomized spontaneously hypertensive female rats (SHR) by reducing oxidative stress. Therefore, the main purpose of this study was to determine the effect of chronic treatment with PHE on the coronary endothelium-dependent vasodilator response.

## Materials and Methods

### Experimental Animals

In this study, 4-weeks-old females SHR (150 ± 10 g body weight) were obtained at the animal facility in the Centre of Health Sciences at Federal University of Espirito Santo. This study was carried out in strict accordance with the recommendations in the Brazilian Guidelines for the Care and Use of Animals for the Scientific Purpose and Didactics and the Guidelines Euthanasia Practice (CONCEA-MCT, 2013). All procedures were approved by the Committee on the Ethics of Animal Care and Use of the Federal University of Espirito Santo under protocol No. 054/2012. The animals were housed in collective cages (five animals per cage) and were provided food (Purina Labina) and water *ad libitum*. Also, they were maintained under controlled conditions of temperature (22–24°C) and humidity (40–60%), with a 12/12 h light-dark cycle. The animals were randomly divided into four groups: Sham Vehicle (Sham-V), Sham Pomegranate hydroalcoholic extract (Sham-PHE), OVX Vehicle (OVX-V), and OVX Pomegranate hydroalcoholic extract (OVX-PHE).

### Ovariectomy

The ovariectomy was performed under general anesthesia with ketamine (80 mg/kg) and xylazine (12 mg/kg) *i.p*. The bilateral dorsolateral incision was made through the skin and underlying muscle was dissected to locate the ovary and uterine tube. The tube was ligated with a suture line and the ovaries were removed. The muscle and skin were then sutured with an absorbable suture. After the surgery animals received an injection of antibiotic (2,5% enrofloxacin, 0,1 mL, *i.m*). In Sham animals, it was made a fictitious surgical procedure (Claudio et al., 2013).

### Preparation of Pomegranate Peel Extract

The plant material of choice (*P. granatum L.*) belongs to the family *Lythraceae*. The plant samples were authenticated by Dr^a^. Valquíria Ferreira Dutra at the Department of Biological Sciences, Federal University of Espirito Santo, where a sample (voucher specimen number 37631) was deposited in the herbarium of the VIES/UFES in botany sector. Extract preparation protocol was modified from that described by Lapornik et al. (2005). The pomegranate was collected for extraction purposes, the peel was removed and dried for 5 days and then grounded. The material (85,71 g) was mixed in 1000 mL of ethanol (95 GL) in an amber bottle until the complete extraction of peel compounds. Afterward, the sample was *vacuum* filtrated, the supernatant was collected, the alcohol was evaporated in a rotary evaporator at 60°C. The crude extract (68%, w/w) was kept in a 4°C in an amber bottle.

### Phytochemical Analysis

Extracts were tested for the presence of active principles such as triterpenoids, steroids, glycosides, saponins, alkaloids, flavonoids, tannins, proteins, free amino acids, carbohydrate, and organic acid. Standard procedures were used (Bhandary et al., 2012).

### Treatment of Pomegranate Peel Extract

The PHE was diluted in filtered water and administered for 30 days orally by gavage (250 mg/kg). The control groups received filtered water as vehicle. The treatment started after 3 days of post-surgical and at the end of the treatment, the animals were 8-weeks-old.

### Non-invasive Assessment of Blood Pressure

After a period of adaptation, the animals were placed in a heated chamber within a container and restrained with a pneumatic cuff attached to the proximal region of the tail. A sphygmomanometer was inflated and deflated automatically, and the systolic blood pressure (SBP) was recorded using a transducer coupled to a computer, as previously described (Baldo et al., 2011). The temperature was maintained at 29 and 32°C for 40 min, during which the animals remained in the chamber (IITC INC/Life Science, 23924 Victory Blvd, Woodland Hills, CA 91367-1253, USA). Averages of three measurements were obtained, with a maximum difference of 10 mmHg, and measurements associated with animal movements were discarded. The non-invasive measurement was done 1 day before the treatment had started and on the last day of the treatment (30th).

### Studies in Isolated Hearts

At the end of treatment, the animals were anesthetized with ketamine (80 mg/kg) and xylazine (12 mg/kg) *i.p* and euthanized via decapitation for blood collection. The heart was excised and immediately transferred to the perfusion apparatus and isolated through cannulation of the aorta. The studies on the coronary vascular bed were performed on whole hearts using a modified Langendorff preparation for perfused isolated hearts as previously described (Santos et al., 2004). Briefly, using a Langendorff apparatus (Hugo Sachs Electronics, March-Hugstetten, Germany), the isolated hearts were perfused with modified Krebs solution containing 120 mM NaCl, 1.25 mM CaCl_2_.2H_2_O, 5.4 mM KCl, 2.5 mM MgSO_4_.7H_2_O, 2.0 mM NaH_2_PO_4_.H_2_O, 27.0 mM NaHCO_3_, 1.2 mM Na_2_SO_4_, 0.03 mM EDTA, and 11.0 mM glucose, heated continuously at 37°C in a water bath and equilibrated with a 95% oxygen and 5% carbon dioxide mixture at a controlled pressure of 100 mmHg to give a pH of 7.4. Coronary flow was maintained constant at 10 mL/min using a roller pump (Hugo Sachs, Germany).

The baseline coronary perfusion pressure (CPP) was measured using a pressure transducer (AD Instruments MLT0380/A Reusable BP Transducer) connected in proximity to the aortic perfusion cannula through which the coronary artery bed was perfused and connected to a digital data acquisition system (PowerLab System). The obtained data were analyzed using LabChart 7.3.1 software (Copyright © 1994–2011 ADInstruments). Because the flow rate was maintained constant at 10 mL/min with a roller pump, the changes in CPP were directly related to changes in vascular resistance. A latex balloon at the end of a steel cannula was inserted into the left ventricle and connected to a pressure transducer (AD Instruments MLT0380/A Reusable BP Transducer) for measurement of the isovolumetric cardiac force. The balloon was pressurized with the aid of a glass syringe to maintain a preload of 10 mmHg.

After stabilization of the system for 40 min, baseline CPP was calculated, and the endothelium-dependent vasodilation was analyzed in coronary arterial bed, randomly, through *in bolus* administration (0.1 mL) of bradykinin (Sigma, St. Louis, MO, USA) in concentrations of 0.1, 1, 10, 100, and 1000 ng, followed by 100 μM of *N*^ω^-nitro-L-arginine ethyl ester (L-NAME; non-specific inhibitor of nitric oxide synthase – NOS). The relaxing response was expressed as percentage of relaxation and calculated by the following equation for each dose:

$$\Delta \%=\left(\frac{{\text{CPP}}_{\text{after infusion}}\times 100}{{\text{CPP}}_{\text{before infusion}}}\right)-100$$

### Isolation of Coronary Arteries

At the end of treatment, the animals were anesthetized with ketamine (80 mg/kg) and xylazine (12 mg/kg) *i.p* and euthanized via decapitation for blood collection. The thorax cavity was opened, and the heart was removed and placed in cold Krebs-Henseleit solution buffer (in mmol/L): 115 NaCl, 25 NaHCO_3_, 4,7 KCl, 1,2 MgSO_4_.7H_2_O, 2,5 CaCl_2_, 1,2 KH_2_PO_4_, 5,5 glucose and 0,01 Na_2_EDTA at pH 7.4 during the dissection procedure. The left anterior descending branch of the left coronary artery and the septal branch were isolated in a dissection microscope (D.F. Vasconcelos M900, São Paulo, Brazil), free of surrounding ventricular muscle tissue and snap frozen in liquid nitrogen. Afterward, the samples were stored at -80°C until their use (Claudio et al., 2013).

### Western Blotting

The hearts were collected, septal coronary arteries dissecated, then, the arteries were quickly frozen and stored until be processed for Western Blot analysis. Each sample was homogenized in modified RIPA buffer (in mmol/L): Tris-HCl 65.2; NaCl 154; NP-40 1% sodium deoxycolate 0.25%; EDTA 0.8; PMSF 1; Sodium orthovanadate 10; Sodium fluoride 100; Sodium Pyrophosphate 10 and protease inhibitor to prevent proteolysis and maintain the protein phosphorylation. Tissue homogenates were centrifuged at 10,000 rpm and 4°C for 10 min to remove tissue debris. Protein concentration in the samples was determined by Bradford method (Quick Start Bradford, Bio-Rad). Protein from the tissue samples (10 μg) was separated on 8% SDS-PAGE and transferred to a nitrocellulose membrane. Membranes were blocked for 120 min with 5% non-fat milk in Tris-buffered solution at room temperature. Membranes were incubated with rabbit primary antibody against p-eNOS Ser^1177^ (1:1,000, Cell Signaling, cat 95715, lot #14, USA), mouse primary antibody against p-eNOS Thr^495^ (1:1,000, BD, cat 612707, lot #2146880, USA), mouse primary antibody against NOS-3.(1:2,000, BD, cat 610296, lot #4077705, USA), rabbit primary antibody against p-AKT Ser^473^ (1:2,000, Cell Signaling, cat 4060, lot #19, USA), rabbit primary antibody against AKT (1:1,000, Cell Signaling, cat 4685, lot #3, USA), mouse monoclonal anti-catalase antibody (1:2000; Sigma, cat. C0979, lot. #082M4837, USA) and a rabbit polyclonal anti-SOD-2 antibody (1:500; Sigma, cat. SAB2102261, lot #QC25874, USA overnight, at 4°C. Afterward, membranes were incubated with a HRP-conjugated goat anti-rabbit (1:10,000, Cell Signaling, cat 7074, lot #24, USA) or horse anti-mouse (1:10,000 Cell Signaling, cat 7076, lot #27, USA) secondary antibody for 60 min at room temperature. Protein bands were identified by means of chemiluminescence (ECL, GE Healthcare) and measured by densitometry.

### Detection of Superoxide Production

The protocol of Dihydroethidium (DHE) staining was performed according to Dos Santos et al. (2016). Briefly, unfixed frozen coronary sections (8 μm) were incubated with 2 μmol DHE (Molecular Probes, Sigma, D7008) in modified Krebs solution containing 20 mmol Krebs-HEPES for 30 min in a light-protected chamber at 37°C. Subsequently, the samples were subjected to wash with phosphate-buffered saline (PBS), and mounted with neutral glycerine. The levels of ROS were determined using microscopy, and coronary fluorescence was quantified with microscope software (Image Pro Plus 4.0). The red fluorescence intensity of the coronaries was quantified in 10 arbitrarily selected coronaries, and the mean value for each islet was calculated.

### Nitrite Measurement in the Plasma

Nitrite (${NO}_{2}^{-}$) concentration was evaluated using the Griess method as described previously with modifications (Silva et al., 2011). Briefly, in a 96 wells plate were added a sample (100 μL) of rat plasma and 100 μL Griess reagent freshly prepared [1% sulfanilamide, 0.1% *N*-(1-naphthyl) ethylenediamine and 2.5% phosphoric acid]. After homogenization, the mixture was incubated for 10 min, at room temperature. The absorbance was measured at 548 nm by spectrophotometry (Epoch Microplate Spectrophotometer, Biotek^®^ , VT, USA) and total nitrite concentrations were calculated using a standard curve build with known concentrations of nitrite. The water was considerate as blank solution.

### Serological Dosages

For determination of the lipid profiles, the blood was centrifuged (Centrifuge Excelsa^®^ IV Model 280R) at 3500 rpm for 15 min at 4°C, and the serum was collected and stored at -20°C. The concentrations of total cholesterol (TC), triglycerides (TG), and high-density lipoprotein (HDL) cholesterol were measured using the enzymatic methods of the Colestat enzyme kit AA, TG Color GPO/PAP AA kit, and HDL cholesterol monophase AA Plus kit, respectively, with a Konelab model 600i spectrophotometer. The concentrations of low-density lipoprotein (LDL) cholesterol and very-low-density lipoprotein (VLDL) cholesterol were calculated using the Friedewald equation (Friedewald et al., 1972): VLDL cholesterol = triglycerides/5, and LDL cholesterol = total cholesterol – (HDL + VLDL).

### Statistical Analysis

All data are expressed as the mean ± SEM. To identify possible outlier data, a two-sided Grubbs’ test was used to identify whether at least one outlier was present in each data set. When the Grubbs’ test identified one outlier, we used an adapted ROUT method to detect any outliers from that column data and removed them according to the Q setting at 1% (alpha = 0.01). For each data set, D’Agostino-Pearson omnibus normality test was also performed. If the data passed the normality test, then comparisons among groups were performed through one-way analysis of variance (one-way ANOVA) followed by Tukey’s *post hoc* test, considering the treatment as the main factor. The vasodilator response to bradykinin was evaluated through two-way analysis of variance (two-way ANOVA) followed by Bonferroni’s *post hoc* test and a significance level of *P* < 0.05 was established.

## Results

### Qualitative Phytochemical Analysis

A qualitative phytochemical analysis was made and the results showed the presence of triterpenoids, glycosides, saponins, flavonoids, tannins, carbohydrates, and organic acid in the PHE (**Table 1**).

**Table 1:** Qualitative phytochemical test of the pomegranate peel hydroalcoholic extract.

Test	Bhandary et al., 2012	PHE
**(I) Test for triterpenes and steroids**		
Liebermann Burchard test	-	DN
Test with chloroform, acetic anhydride and H_2_SO_4_	DN	+
**(II) Test for glycosides**		
Keller Killiani test	-	DN
Bromine water test	-	DN
Test with Sodium nitroprusside 5%	DN	+
Baljet reactive	DN	+
**(III) Test for Saponins**		
Foam test	-	+
**(IV) Test for Alkaloids**		
Hager’s Test	-	DN
Bouchardat reactive	DN	-
Dragendorff reactive	DN	-
**(V) Test for Flavonoids/Phenols**		
FeCl_3_ test	+	+
Test with alkaline reagent	+	+ (Flavonoids)
Test with acid reagent	DN	-
Test with acetate solution	+	DN
**(VI) Test for Tannins**		
Gelatin test	+	DN
Test with FeCl_3_	DN	+
**(VII) Test for protein**		
Biureto test	-	-
Spectroscopy	DN	-
**(VIII) Test for free amino acids**		
Test with ninhydrine	-	-
**(IX) Test for Carbohydrates**		
Benedict test	+	DN
Test with lugol	DN	-
Reducing sugars (Fehling reactive)	DN	+
Sugar. not Reducers/heterosides (Fehling reactive)	DN	+
**(X) Test for Vitamin C**		
DNPH test	-	DN
**(XI) Test for Organic acids**		
Pascová reactive	DN	+


*DN, did not; +, present;-, not present.*

### Effects of PHE on Systolic Blood Pressure

**FIGURE 1:**
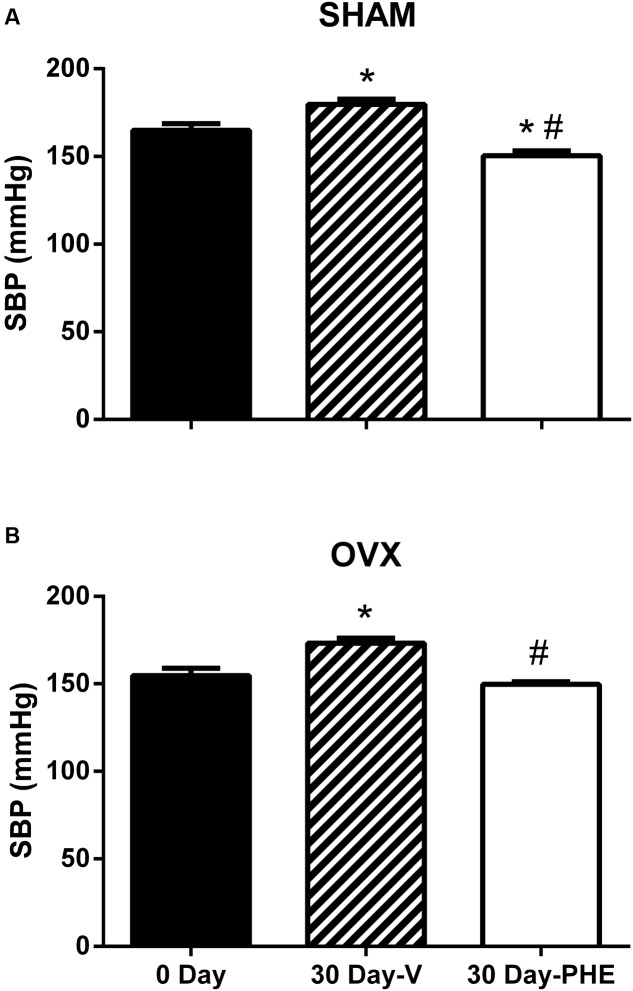
**Systolic blood pressure (SBP) at the beginning (0 day) and end of treatment (30 days).** Vehicle (V) and Pomegranate hydroalcoholic extract (PHE) in Sham group **(A)** and Ovariectomized group (OVX) **(B)**. Values are expressed as mean ± SEM; *n* = 10–16 animals per group. ^∗^*p* < 0.05 compared to 0 Day values, ^#^*p* < 0.05 compared to the Vehicle group.

The SBP data are summarized in **Figures 1A,B**. As expected, 30 days after the beginning of the study, we observed that the SBP increased in Sham-V group (165 ± 4 to 180 ± 3 mmHg, *P* < 0.05) as well as in OVX-V (155 ± 4 to 173 ± 3 mmHg, *P* < 0.05). In addition, we found that the treatment with PHE decreases SBP in Sham-PHE group (150 ± 3 mmHg, *P* < 0.05). In the other hand, the treatment was able to prevent the progression of hypertension in OVX-PHE (150 ± 1.5 mmHg, *P* < 0.05) group when compared to the beginning of the treatment.

### Coronary Vascular Reactivity

**FIGURE 2:**
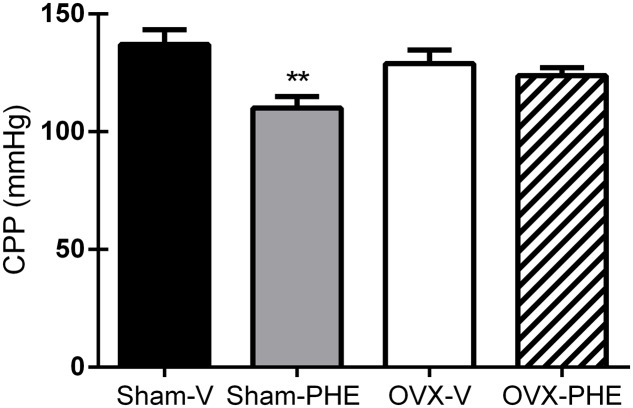
**Baseline coronary perfusion pressure (CPP) after the treatment.** Sham treated with Vehicle (Sham-V), Sham treated with Pomegranate hydroalcoholic extract (Sham-PHE), Ovariectomized treated with Vehicle (OVX-V) and Ovariectomized treated with Pomegranate hydroalcoholic extract (OVX-PHE) groups. Values are expressed as mean ± SEM; *n* = 9–12 animals per group. ^∗∗^*p* < 0.01 compared to the Sham-V group.

**FIGURE 3:**
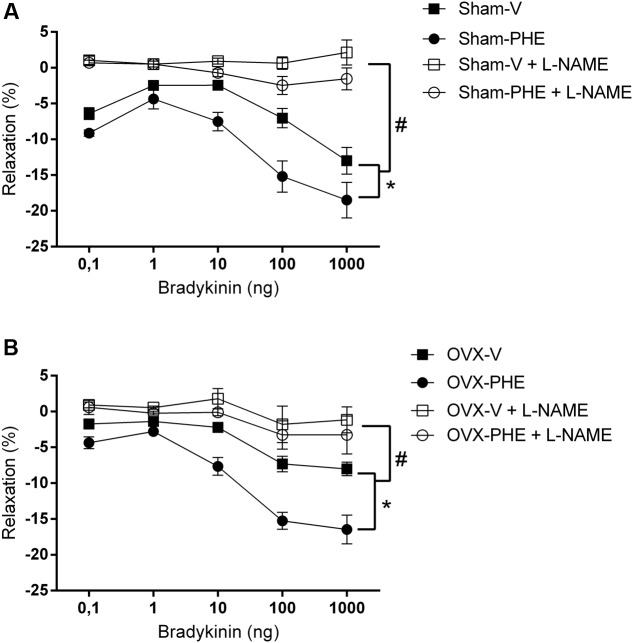
**Dose-response curves to bradykinin (0,1–1000 ng) in isolated hearts.** Sham group **(A)** and OVX group **(B)** before (closed symbols) and after (open symbols) perfusion with L-NAME (100 μM) for 20 min. Data were expressed as mean ± SEM; *n* = 6–10 animals per group. ^∗^*p* < 0.05 compared with the vehicle group before L-NAME. ^#^*p* < 0.05 compared with the both groups after L-NAME.

As showed in **Figure 2**, we observed that the treatment with PHE significantly reduced the basal CPP in the Sham group (Sham-V: 137 ± 6 mmHg vs. Sham-PHE: 110 ± 5 mmHg, *P* < 0.01). In **Figure 3**, we analyzed the functionality of the endothelium through the curve dose-response to bradykinin and observed that the vasodilator response was present in both the Sham (**Figure 3A**) and OVX (**Figure 3B**) groups. The treatment significantly increased vasodilator response when compared to their respective control groups (*P* < 0.05). After inhibition with L-NAME, we observed an attenuation of the vasodilator response in all groups (*P* < 0.05).

### Analysis of Protein Expression (Western Blotting)

**FIGURE 4:**
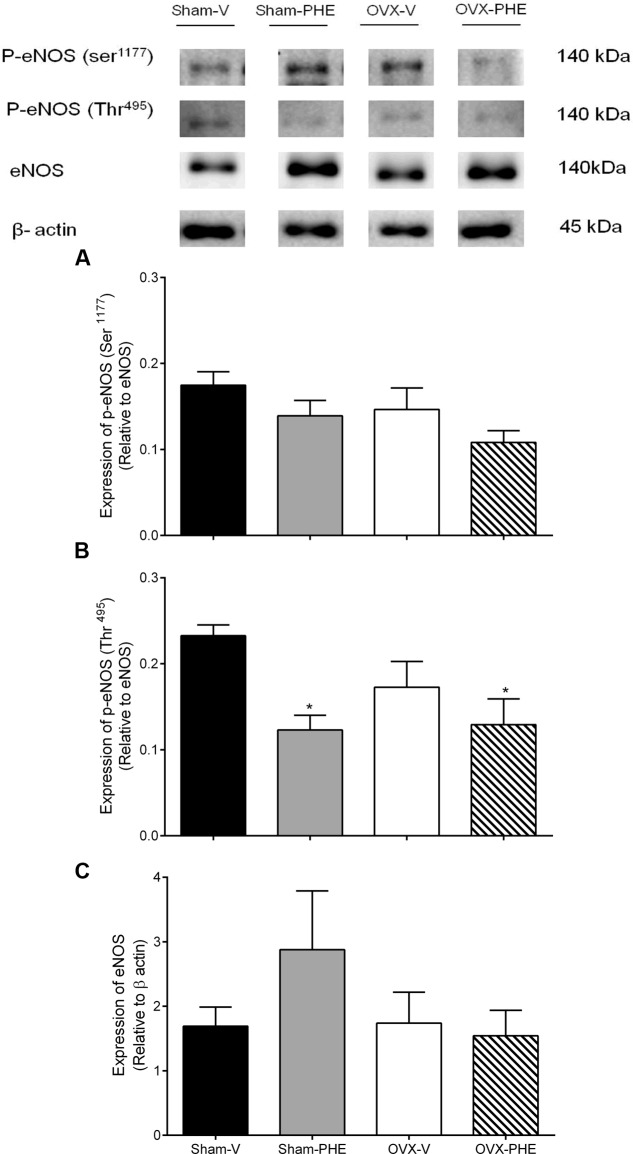
**Analysis of protein expression in Sham and OVX rats.** **(A)** p-eNOS (Ser^1177^), **(B)** p-NOS (Thr^495^) and **(C)** total eNOS in Sham-V, Sham-PHE, OVX-V and OVX-PHE. Each sample was obtained from 1 vessel from coronary septal arteries, and the analyses of different proteins were made in the same gels using isolated strip buffer. However, the organization of the groups not allowed us to remove a representative image of the blots in a single gel. Thus, for better clarification we reorganized the blots separately. Data were expressed as mean ± SEM; *n* = 5–10 per group. ^∗^*p* < 0.05 compared to Sham-V group.

**FIGURE 5:**
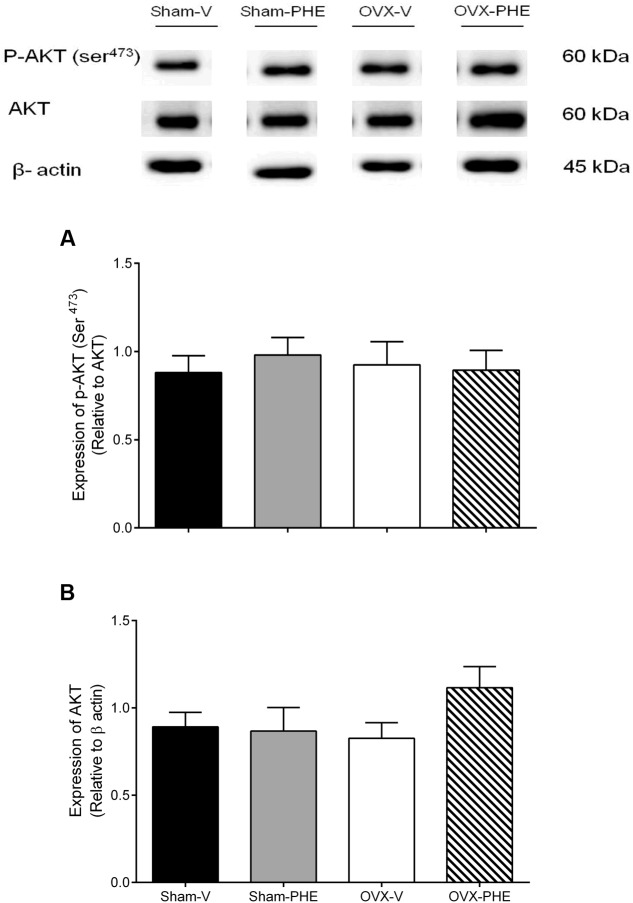
**Analysis of protein expression in Sham and OVX rats.** **(A)** p-AKT (Ser ^473^), **(B)** total AKT in Sham-V, Sham-PHE, OVX-V, and OVX-PHE. Each sample was obtained from 1 vessel from coronary septal arteries, and the analyses of different proteins were made in the same gels using isolated strip buffer. However, the organization of the groups not allowed us to remove a representative image of the blots in a single gel. Thus, for better clarification we reorganized the blots separately. Data were expressed as mean ± SEM; *n* = 5–10 per group.

**FIGURE 6:**
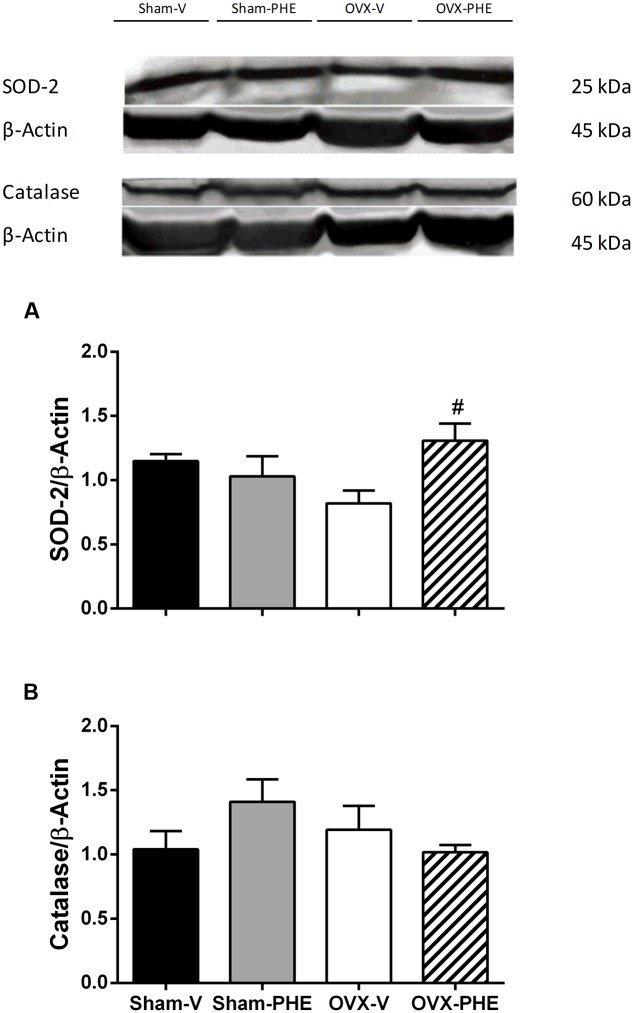
**Analysis of protein expression in Sham and OVX rats.** **(A)** superoxide dismutase, SOD-2 and **(B)** catalase in Sham-V, Sham-PHE, OVX-V, and OVX-PHE. Data were expressed as mean ± SEM; *n* = 4–7 per group. ^#^*p* < 0.05 compared with OVX-V group.

We found that the treatment was able to decrease the p-eNOS (Thr^495^) in Sham (Sham-V: 0.23 ± 0.01 vs. Sham-PHE: 0.12 ± 0.02, *P* < 0.05) and OVX groups (OVX-V: 0.19 ± 0.03 vs. OVX-PHE: 0.12 ± 0.03, *P* < 0.05) (**Figure 4B**). However, we did not observe significant differences in the p-eNOS Ser^1177^ (**Figure 4A**), total eNOS (**Figure 4C**), p-AKT Ser^473^ (**Figure 5A**) and total AKT (**Figure 5B**). In addition, the PHE was effective to prevent the decreasing in expression of enzymes SOD-2 (**Figure 6A**) in the OVX group (OVX-V: 0.82 ± 0.1 vs. OVX-PHE: 1.31 ± 0.1, *P* < 0.05) and not changed the catalase expression in any group (**Figure 6B**).

### Analysis of the Superoxide Anion

**FIGURE 7:**
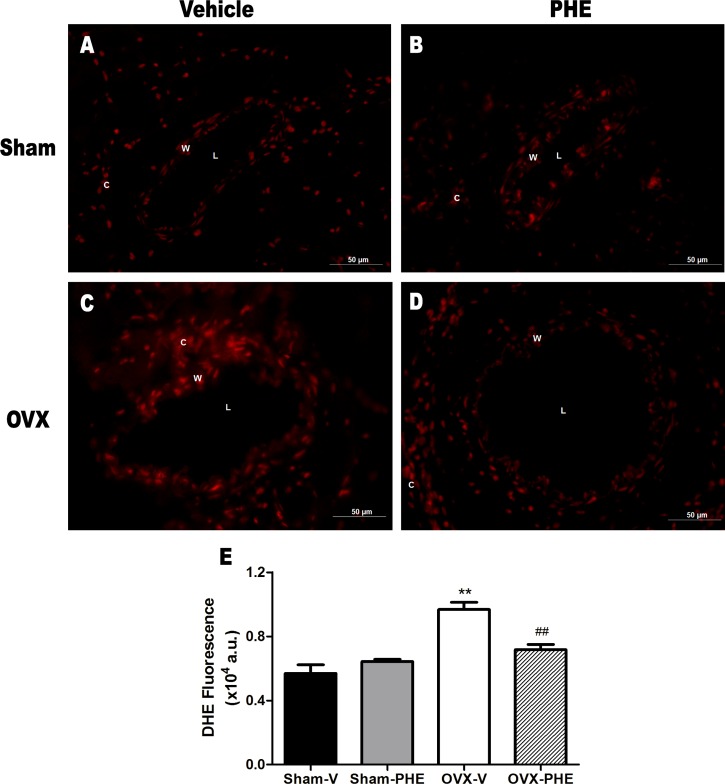
**Influence of pomegranate peel extract treatment on superoxide anion production in coronary arteries.** The top panels **(A–D)** show higher fluorescence intensity (red) in OVX-V rats when compared both Sham and OVX-PHE rats using dihydroethidium (DHE) staining. Left panel, vehicle treatment and right panel, pomegranate hydroalcoholic extract treatment (PHE). **(E)** The bar graph shows the average DHE fluorescence (AU: arbitrary units) comparing all groups (*n* = 9–12). Values are means ± SEM. ^∗∗^*p* < 0.01 compared with Sham, and ^##^*p* < 0.01 compared with OVX-PHE group. Scale bar: 50 μm.

The DHE oxidative assay revealed intense fluorescence in OVX-V group when compared both Sham-V and OVX-PHE animals. Interestingly, the antioxidant effect of the treatment was observed in the OVX-PHE coronary arteries group, as represented in **Figure 7**. (Sham-V: 0.57 ± 0.05 vs. Sham-PHE: 0.64 ± 0.01 vs. OVX-V: 0.96 ± 0.04 vs. OVX-PHE: 0.76 ± 0.03, *P* < 0.01).

### Analysis of Plasma Nitrite Levels

**FIGURE 8:**
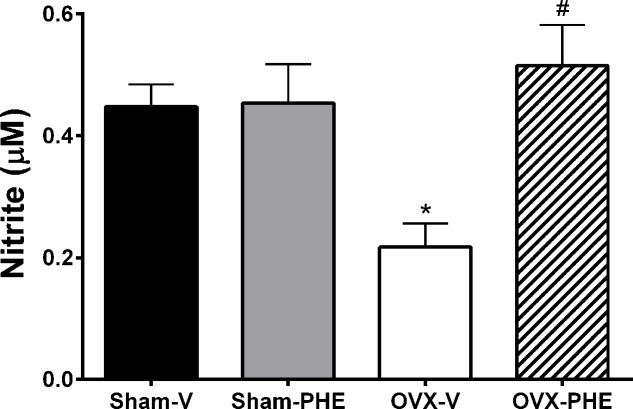
**Analysis of plasma nitrite levels.** Values in Sham-V, Sham-PHE, OVX-V and OVX-PHE. Data were expressed as mean ± SEM; *n* = 6–12 animals per group. ^∗^*p* < 0.05 compared to the Sham-V group. ^#^*p* < 0.05 compared with OVX-V group.

The treatment did not increase the plasma nitrite formation in Sham-PHE group (Sham-V: 0.448 ± 0.04 μM and Sham-PHE: 0.454 ± 0.06 μM) (**Figure 8**). On the other hand, the hormonal dysfunction induced by castration reduced the formation of plasma nitrite (Sham-V: 0.448 ± 0.04 μM vs. OVX-V: 0.212 ± 0.04 μM, *P* < 0.05). The treatment in OVX group, in turn, was able to prevent the reduction in plasma nitrite levels (OVX-V: 0.212 ± 0.04 μM vs. OVX-PHE: 0.515 ± 0.07 μM, *P* < 0.05).

### Analysis of Lipid Profile

We observed a significant reduction in the levels of total cholesterol and LDL (**Table 2**) in the Sham-PHE group compared to the Sham-V group. In the OVX-PHE group, the treatment significantly reduced the levels of LDL and triglycerides when compared to the OVX-V. HDL concentration was not altered in any of the studied groups.

**Table 2:** Analysis of lipid profile in Sham and ovariectomized (OVX) rats (*n* = 7–13 animals per group).

Groups	TC	TG	HDL	LDL
	(mg/dL)	(mg/dL)	(mg/dL)	(mg/dL)
Sham-V	54 ± 5.4	54 ± 5.6	15.6 ± 0.6	21 ± 1.5
Sham-PHE	39 ± 2.9^∗∗^	74 ± 6.8	15.8 ± 0.7	14 ± 0.6^∗^
OVX-V	54 ± 1.4	65 ± 4.5	15.7 ± 0.7	24 ± 1.7
OVX-PHE	48 ± 1.6	37 ± 3.1^#^	16.3 ± 0.5	18 ± 1.5^#^


*Data were expressed as ±SEM. ^∗^*p* < 0.05, ^∗∗^*p* < 0.01 compared to Sham-V group. ^#^*p* < 0.05 compared to OVX-V group.*

*TC, total cholesterol; TG, triglycerides; HDL, high-density lipoprotein and LDL, low-density lipoprotein.*

## Discussion

The main finding of the present study is that the treatment with PHE was able to improve the endothelium-dependent coronary relaxation and these effects can be related to inhibition of eNOS phosphorylation at its inhibitory residue Thr^495^ and reduce oxidative stress. In addition, the treatment was effective in attenuating the SBP and in preventing its progression.

As expected, 30 days after the beginning of the study we observed that the SBP increased in both groups (Sham-V and OVX-V). This result has been showed by other studies (Okamoto and Aoki, 1963; Imada et al., 1985; Androwiki et al., 2015). Moreover, we found that the treatment with PHE decreased SBP in Sham-PHE group. In addition, even that the treatment was not able to reduce the SBP in OVX-PHE group when compared to the onset of the treatment, we could observe that it prevented the progression of hypertension. A possible explanation for our findings is related to the fact that PHE can act as a therapeutic agent in hypertension due to its potent antioxidant activity, via polyphenols. These polyphenols are capable of protecting nitric oxide (NO) against superoxide anion ($O_{2}^{•-}$) – mediated oxidation, thus increasing its biological actions, such as endothelium-dependent relaxation (Ignarro et al., 2006). There is a relationship between hypertension and impaired endothelial function, leading a decrease in NO bioavailability, which characterizes the endothelial dysfunction (Hamilton et al., 2001). In hypertension occurs an increase in $O_{2}^{•-}$ generation leading to a lower NO bioavailability. $O_{2}^{•-}$ rapidly reacts with NO, forming peroxynitrite and decreasing NO bioavailability (Rey et al., 2001; Androwiki et al., 2015). Additionally, sex hormonal deficiency enhances the progression of endothelial dysfunction and studies have indicated that the cardiovascular protection observed in premenopausal females has been attributed to the beneficial effects of estrogen on endothelial function (Reckelhoff, 2001). estrogen can act on releasing of endothelium-derived vasodilator factors and also inhibit the renin–angiotensin system (Reckelhoff et al., 2000; Santos et al., 2010).

Pomegranate has a high antioxidant capacity due to raised polyphenols concentrations, more specifically hydrolysable tannins, which are capable of scavenging free radical (Aviram and Rosenblat, 2012). Besides its scavenger effect, we have previously shown that pomegranate is able to reduce the angiotensin-converting enzyme (ACE) activity (Santos et al., 2016) contributing to an environment with less oxidative stress consequently reducing CVDs risk (Aviram and Dornfeld, 2001; Mohan et al., 2010). In addition, with the ACE inhibition there is a decreasing in bradykinin degradation that may contribute to our results (Shiuchi et al., 2002).

We observed that the treatment was effective in reducing the baseline CPP only in the Sham-PHE group. Hormonal dysfunction in OVX females is an important factor to the development of hypertension and coronary artery disease because the presence of estrogen is able to modulate the NO synthesis by acting on the isoforms of the enzyme nitric oxide synthase (NOS). Indeed, results from our laboratory indicate that the main endothelial mediator responsible for regulating the coronary vascular tone (represented by the baseline CPP) is the NO in both Wistar and SHR rats (Santos et al., 2004, 2010, 2016). Therefore, ovariectomy leads to damages on the endothelial function, as previously showed by our study group (Borgo et al., 2011; Caliman et al., 2013; Claudio et al., 2013; Oliveira et al., 2014; Lamas et al., 2015); impairing thus, the preventive/therapeutic effects from the PHE on the baseline CPP values in the OVX-PHE group. Another suggestion is that the beneficial effects from the treatment are probably dependent on circulating estrogen levels in the blood, since we observed that animals with normal estrogen levels (Sham) showed the benefits generated by the treatment. In view of this hypothesis, we suggest that the castration may have caused a reduction in the beneficial effects of estrogen, perhaps by reduction of its receptors in coronary arteries of the hypertensive rats, as previously showed (Borgo et al., 2016).

Such antioxidant characteristics of polyphenols confer protective effects on the blood vessels due to a reduction of oxidative stress in endothelial cells, improving the vascular reactivity. Thus, our results are in agreement with literature data, since the endothelium-dependent relaxation was enhanced in all treated groups (Machha and Mustafa, 2005; Ajay et al., 2007; Yilmaz and Usta, 2013). Nevertheless, L-NAME impaired the endothelium-dependent relaxation, which shows that there is a strong participation of NO in this response. To our knowledge, we are the first ones to demonstrate the PHE treatment modulates vascular reactivity of the coronary bed. Our data corroborate the study of Machha and Mustafa (2005), which showed that chronic treatment with flavonoids improves endothelium-dependent relaxation, reduces SBP and significantly improves endothelial function in hypertensive animals. The PHE acts as exogenous antioxidant system neutralizing ROS and increases the NO bioavailability. It is known that the antioxidant system is any substance that, when present at low concentration compared with those of any oxidizable substrate, significantly delays or prevents oxidation of that substrate (Halliwell and Gutteridge, 2007).

Addition to the elevated concentrations of polyphenols with high antioxidant capacity, the pomegranate has a high concentration of phytoestrogens which can confer cardioprotective effects similar to estrogen. Among the phytoestrogens that are known, urolithins A and B (enterophytoestrogens), which are microflora metabolites from the ellagic acid that belongs to the tannins class, display both oestrogenic and antioestrogenic activities (Larrosa et al., 2006; Ryu et al., 2016). It is know that estrogen phosphorylate eNOS mainly via the serina (Ser)/threonine (Thr) kinase Akt (Förstermann and Sessa, 2012). In fact, the treatment promotes its beneficial effects through the inhibition of eNOS phosphorylation at its inhibitory residue Thr^495^. However, we not observed alterations after the treatment in total eNOS and total AKT as well as in their phosphorylated form in residue Ser^1177^. Another Therefore, the PHE effect that may have contributed to improved endothelium-dependent relaxation may be related to its antioxidant properties described in the literature (Gil et al., 2000) and its scavenging capacity (Sun et al., 2016). It was postulated that treatment with PHE might decrease oxidative stress in animals with hypertension (Dos Santos et al., 2016). It is know that the oxidative stress is related to an increased ROS generation. Such increase overloads the antioxidant system to neutralize these substances, which can induce the development and/or progression of chronic degenerative diseases such as hypertension and coronary heart disease (Beswick et al., 2001; Shah and Channon, 2004; Androwiki et al., 2015).

In view of this, we decided to analyze the ability of treatment in reducing oxidative stress by the DHE oxidative assay. We observed an intense fluorescence in OVX-V group when compared with both Sham-V and OVX-PHE animals. In addition, the treatment was able to reduces the $O_{2}^{•-}$ production. These results support the notion that one of the mechanisms involved in attenuation of damage derived from hypertension may be related to the reduction of oxidative stress and increasing of the NO bioavailability as demonstrated in this study. This result corroborates a previous study of from our laboratory (Dos Santos et al., 2016). Thus, the cardioprotective effects from PHE observed in this study can be in part derived from antioxidant action of the pomegranate. Indeed, in our study, a qualitative phytochemical screening of the crude extract revealed the presence of antioxidants substances, i.e., tannins and flavonoids.

The most important finding in this study was the capacity of treatment with PHE in improving coronary vascular reactivity. We believe that the increased endothelium-dependent relaxation and the reduction in the level of $O_{2}^{•-}$ in the Sham-PHE group suggest that the treatment reduces ROS concentration and increases NO bioavailability. We also investigated whether the treatment with PHE was able to alter the expression of antioxidant enzymes such as SOD-2 and catalase. Interestingly, our data showed that after the treatment, there was a significant increase in the SOD-2 expression in the OVX-PHE group, enzyme that promotes $O_{2}^{•-}$ dismutation, which is formed in greater amounts in hypertension (Androwiki et al., 2015). Our results corroborate data from the literature showing the relationship between pomegranate and antioxidant system activity, in which polyphenois promoted upregulation in SOD in hypertensive rats (Bagri et al., 2009; Althunibat et al., 2010; Mohan et al., 2010).

Our results corroborate Ignarro et al. (2006) that showed that the treatment with pomegranate promotes an increase in the biological actions of NO by antioxidant effects of polyphenols and not only through changes in transcriptional expression or catalytic activity of eNOS. Therefore, we believe that the treatment with PHE prevented the reduction of NO bioavailability induced by castration through: (i) its antioxidant action and (ii) increase in the SOD-2 expression. These actions in conjunction with the increase in the generation of plasma nitrite levels could explain the results found in this study.

Phytoestrogens have a function similar to estrogen, such as hypocholesterolemic effects, that could reduce the risk of CVDs associated with menopause (Lissin and Cooke, 2000; Gencel et al., 2012). As expected, we observed an improvement in lipid profile in both treated groups. Indeed, other studies showed that pomegranate extract (PHE) improves atherogenic markers (Neyrinck et al., 2013). These results were also observed in clinical studies, where the consumption of concentrated pomegranate juice improved lipid profile (Esmaillzadeh et al., 2004). Fuhrman et al. (2010) demonstrated that the polyphenols in pomegranate can increase levels of the enzyme HDL-associated PON-1 and reduced LDL-associated PON-1. The PON-1, also known as aromatic esterase 1, is responsible for protecting HDL and LDL of lipid oxidation.

## Conclusion

We demonstrated that the use of PHE has beneficial effects contributing to: (i) reduction in SBP; (ii) increase in the endothelium-dependent relaxation in coronary arteries; (iii) reduction in oxidative stress; (iv) inhibition of eNOS phosphorylation at its inhibitory residue Thr^495^ and (v) improve in lipid profile. Taken together, these effects can protect against the development and/or progression of CVDs. Most of these effects may be related, at least in part, with estrogenic actions of phytoestrogens. These findings have important implications for the prevention of CVDs, especially in coronary vascular bed of the female with sex hormonal deficiency and hypertension. Therefore, the characterization of PHE role may provide a new approach to the development of new forms of therapeutic intervention in the treatment of hypertension associated with hormonal dysfunction.

## Author Contributions

Substantial contributions to the conception or design of the work: ND, WR, RS and MM. The acquisition, analysis: ND, WR, LF-L, TP, AD, JS, AS, RS, and MM. Interpretation of data for the work: ND, WR, LF-L, TP, AD, JS, VL, HM, RS, and MM. Drafting the work: ND, WR, RS, and MM. Revising it critically for important intellectual content: ND, WR, LF-L, TP, AD, JS, VL, AS, HM, RS, and MM. Final approval of the version to be published: ND, WR, RS, and MM. Agreement to be accountable for all aspects of the work in ensuring that questions related to the accuracy: ND, WR, LF-L, TP, AD, JS, VL, AS, HM, RS, and MM integrity of any part of the work are appropriately investigated and resolved: ND, WR, LF-L, TP, AD, JS, VL, AS, HM, RS, and MM.

## Conflict of Interest Statement

The authors declare that the research was conducted in the absence of any commercial or financial relationships that could be construed as a potential conflict of interest.

## Abbreviations

OVX: ovariectomized rats
PHE: pomegranate hydroalcoholic extract
